# Changes in Ion Concentrations upon the Binding of Short Polyelectrolytes on Phospholipid Bilayers: Computer Study Addressing Interesting Physiological Consequences

**DOI:** 10.3390/polym14173634

**Published:** 2022-09-02

**Authors:** Tomáš Blovský, Karel Šindelka, Zuzana Limpouchová, Karel Procházka

**Affiliations:** 1 The Department of Physical and Macromolecular Chemistry, Faculty of Science, Charles University in Prague, Hlavova 2030, 128 40 Prague 2, Czech Republic; 2 Department of Molecular and Mesoscopic Modelling, Institute of Chemical Process Fundamentals of the Czech Academy of Sciences, v.v.i., Rozvojová 135/1, Suchdol, 165 02 Prague 6, Czech Republic

**Keywords:** lipid membrane, antimicrobial peptides, computer simulations, dissipative particle dynamics, entropy of counterions

## Abstract

This computer study was inspired by the experimental observation of Y. Qian et al. published in ACS Applied Materials and Interfaces, 2018 that the short positively charged *β*-peptide chains and their oligomeric analogues efficiently suppress severe medical problems caused by antimicrobial drug-resistant bacteria despite them not penetrating the bacterial membrane. Our coarse-grained molecular dynamics (dissipative particle dynamics) simulations confirm the tentative explanation of the authors of the experimental study that the potent antimicrobial activity is a result of the entropically driven release of divalent ions (mainly magnesium ions essential for the proper biological function of bacteria) into bulk solution upon the electrostatic binding of *β*-peptides to the bacterial membrane. The study shows that in solutions containing cations Na^+^, Ca^2+^ and Mg^2+^, and anions Cl^−^, the divalent cations preferentially concentrate close to the membrane and neutralize the negative charge. Upon the addition of positively charged oligomer chains (models of *β*-peptides and their analogues), the oligomers electrostatically bind to the membrane replacing divalent ions, which are released into bulk solvent. Our simulations indicate that the entropy of small ions (which controls the behavior of synthetic polyelectrolyte solutions) plays an important role in this and also in other similar biologically important systems.

## 1. Introduction

Even though the phospholipid bilayers are relatively simple self-assemblies as compared with a number of other functional biological structures, they belong to the most important constructs in nature. They separate living cells from their surroundings and from each other, and simultaneously they intermediate the communication of cells with each other and with the outside world, which is essential for their life cycles and function, including proliferation and apoptosis.

Phospholipid membranes are inevitable parts of the cells of all living creatures ranging from bacteria to mammals. Individual membranes slightly differ in their mission, function and structure, and the chemical composition and the architecture of the phospholipid molecules that form them also varies. The membranes are often decorated by specific substituents and oligomeric motifs. They host a number of important receptors and channels for passive and active transport of various compounds which secure the proper function of cells. Due to the importance of membranes for life on the Earth, they have been amply studied by biologists, chemists and physicists and the most important features of their behavior have been described in detail in a number of textbooks [1].

Despite a broad variety of functional membranes, their self-assembly and structure are controlled by the same physicochemical principles. All of them are results of a spontaneous self-assembly of amphiphilic compounds in aqueous media, i.e., results of an enthalpically driven process minimizing the number of unfavorable contacts of hydrophobic groups with water. As explained by Nagarajan and others more than three decades ago [2,3,4], enthalpy is a driving force, but the association number and other properties of most assemblies of amphiphiles in selective solvents are controlled by entropy. In the case of amphiphiles in aqueous media, the situation is more complex than that in selective organic solvents, because the association-segregation process involves changes in water structure and in numbers of hydrogen bonds in the vicinity of hydrophobic–hydrophilic interfaces [5]. Due, in part, to a significant asymmetry of hydrophobic and hydrophilic parts and to a relatively high stiffness of relatively short hydrocarbon chains, the assembly process leads to the formation of 2D bilayers with hydrocarbon chains inside and hydrophilic groups at both surfaces in contact with water [6]. An overwhelming majority of cellular membranes contain ionized groups (mainly anionic) which means that their surfaces are non-negligibly negatively charged and the electric charge is compensated by the cloud of closely located mobile counterions, both singly and multiply positively charged. A number of studies show that the proper biochemical function of membranes and particularly the function of imbedded receptors require the presence of calcium and magnesium ions in their immediate vicinity [7,8,9,10,11,12,13].

A few years ago, an interesting example of the effect of double charged calcium and magnesium ions on the function of cell membranes of gram-negative bacteria was published by Qian et al. [14]. Inspired by earlier studies on the host defense peptides [15,16,17,18,19,20,21,22,23,24,25,26,27,28,29] and particularly by studies on β-peptides, [30,31,32,33,34,35,36] the authors studied the possibility to suppress health problems caused by the antimicrobial drug-resistant bacteria by positively charged synthetic analogues of short β-peptides. The β-peptides and their synthetic analogues exhibit a potent antimicrobial activity but the mechanism of their action has not been fully understood. It has been demonstrated that long β-peptide chains penetrate the outer membrane of *Escherichia coli* (*E. coli*) which triggers the disruption of the inner membrane and kills the bacteria [37] but the shorter chains, which are not supposed to translocate across the membrane, have also been found to be fairly active. To prevent the penetration of β-peptide chains through the cell membrane, the authors prepared a brush of relatively short synthetic analogues of β-peptides covalently tethered to the gold surface and incubated *E. coli* and methicillin-resistant *Staphylococcus aureus* (MRSA) on this surface-modified substrate. They found that the undesirable medical problems were considerably suppressed which they attributed to the generic (unspecific) effect of the entropically favorable release of fairly mobile calcium and magnesium ions in bulk solvent upon the electrostatic binding of cells to the oppositely charged brush of surface-tethered β-peptides. To support their explanatory hypothesis, the authors increased the bulk concentration of the double charged calcium and magnesium ions and observed ca. 80% recovery of the β-peptide-treated-bacteria activity.

As the smart polymer-modified surfaces offer the advantageous treatment of the bacteria-generated medical problems, the paper by Qian attracted the interest of a number of research groups. In the last two years, it has been cited more then eighty times and it is futile to try to cite here all important studies; hence we mention only two relevant recent papers [38,39]. Unfortunately, the overwhelming majority of studies focused on the specific biochemical interactions and the generic entropy-driven effect of the ions released into bulk solution escaped from the mainstream of interest of broader scientific community.

Motivated by the observations by Qian et al. and by their tentative explanation, we performed an extensive simulation study aimed at the reported behavior and at the proof of their working hypothesis. The goal of the study is not the accurate emulation of their experiments and reproduction of their results. They were obtained in biological systems under complex conditions and we cannot preclude that they were partly affected by specific effects. We aim at general principles of the behavior and investigate how much the electrostatic binding of multiply charged oligomers to the slightly charged membranes affects the distribution of monovalent and divalent ions in their immediate vicinity, which, in the case of positive results, supports the explanation proposed by Qian et al. While the effect of monovalent ions on the electrostatic binding of peptides at the phospholipid membrane has already been theoretically studied [40], to the best of our knowledge, the changes in the preferential binding of ions differing in valency upon the peptide binding have never been theoretically investigated.

## 2. Simulation Method

In this study, we used the dissipative particle dynamics, DPD (a coarse-grained variant of the generic molecular dynamics method), developed by Hoogerbrugge and Koelman [41] and further modified by Groot, Warren, Español and others [42,43,44], which is a suitable method for studying large systems of many interacting particles. In DPD, the solvent and low-molar-mass compounds are represented by clusters of several molecules (DPD beads). The polymers and oligomers are composed of several interconnected beads. The computational machinery consists of the numerical solution of the Newtonian equation of motion of DPD beads subjected to forces which involve three contributions: (i) a soft conservative force, $F^{C}$, describing the interaction between the coarse-grained beads; (ii) a dissipative force, $F^{D}$, reflecting the friction which slows down the average motion of beads; and (iii) a random force, $F^{R}$, emulating thermal agitation and intermolecular collisions which accelerate the particles. The conservative forces, $F^{C}=-\nabla u^{\mathrm{CG}}$, derive from the coarse-grained inter-bead potentials, $u^{\mathrm{CG}}$. The forces $F^{D}$ and $F^{R}$ substituting high numbers of degrees of freedom neglected by the coarse-graining approach in favor of fast and smooth simulations have to be well balanced to secure constant temperature during the simulation run. Español and Warren [43] addressed this problem and derived a formula (dissipative–fluctuation theorem) which serves as a thermostat and secures a correct distribution of bead velocities by the Maxwell–Boltzmann distribution function at a given temperature. Moreover, the pair-wise nature of dissipative and random forces guarantees that the momentum is conserved locally which in turn ensures the correct hydrodynamic behavior.

Because the DPD approach employs effective forces acting between larger parts of the system (coarse-grained beads), the pair interactions between beads *i* and *j* are “soft” and do not diverge at short distances $r_{ij}$, which means that the DPD beads also behave as “soft” objects and can interpenetrate each other. The conservative forces include: (i) the soft and spatially-limited non-electrostatic repulsion; (ii) electrostatic interaction acting between the charged particles (ions, polyelectrolyte beads, etc.); and (iii) bonding forces, if the polymers, surfactants or larger molecules (e.g., porphyrins) are studied [45]. The soft non-electrostatic repulsion is commonly expressed as:

(1) $$u_{ij}^{sr}=\left\{\begin{array}{cc} \frac{a_{ij}}{2}r_{c}{\left(1-\frac{r_{ij}}{r_{c}}\right)}^{2}\; & \mathrm{for}\;r_{ij}<r_{c}\\ 0 & \mathrm{for}\;r_{ij}\geq r_{c} \end{array}\right.,$$

where $a_{ij}$ is the maximum repulsion between particles *i* and *j*, $r_{c}$ is the interaction cutoff.

Flexible polymer chains are usually modeled as strings of coarse-grained particles connected by elastic springs emulating the covalent bonds. Most often the harmonic spring potential is used to describe the bond strength and elasticity:

(2) $$u_{i,i+1}^{\mathrm{hs}}=\frac{k}{2}{\left(r_{i,i+1}-r_{0}\right)}^{2},$$

where *k* is the spring constant and $r_{0}$ is the equilibrium distance. In contrast to less coarse-grained bead-spring models which employ the Lennard–Jones potential for describing the dispersion force [46], the soft repulsion also acts between two directly bonded beads *i* and i+1 and competes with the spring elasticity. Groot and Warren have shown that values of $k/(k_{B}T$) ($k_{B}$ is the Boltzmann constant and *T* is the thermodynamic temperature) between 2 and 4, together with $r_{0}=0$, prevent excessive bond stretching and are convenient for modeling real polymers. However, some authors use high *k* and non-zero $r_{0}$; the others use different potentials, either FENE or the Morse potential [47]. In the case of semiflexible chains, the angular (cosine) potentials between two bonds or “dihedrals” potentials are also used [45]. An interesting alternative aimed at the bond stretching proposed by Neimark et al. [48] consists of the application of harmonic potentials with relatively large $r_{0}$ between the *i* and (i+2) and possibly also (i+3) beads.

Groot and Warren [42] mapped the DPD results onto Flory–Huggins (FH) systems and established a link between the DPD parameters of soft repulsive forces $a_{ij}$ and the FH interaction parameter $\chi_{ij}$ which describes the interaction of FH segments:

(3) $$\chi_{ij}=2\alpha \rho r_{c}^{3}\left(a_{ij}-a_{ii}\right)\frac{r_{c}}{k_{B}T},$$

where ρ is the total particle density and α is proportional constant dependent on ρ. Using the appropriate equation of state for the soft repulsive DPD fluid together with the value of compressibility for ambient water, and assuming $a_{ii}=a_{jj}$, they derived for polymer systems at $\rho r_{c}^{3}=3$ the following simple relation

(4) $$\frac{a_{ij}r_{c}}{k_{B}T}=\frac{a_{ii}r_{c}}{k_{B}T}+3.27\chi_{ij}.$$

We would like to remind the reader that the favorable interactions between the components, described by χ=0 translate into $a_{ij}=25$, the value $a_{ij}=26.64$ corresponds to the θ-state (χ=1/2) and $a_{ij}=40$ describes strongly unfavorable interactions corresponding to χ=4.59.

If some components of the studied system are electrically charged, the electrostatic interactions represent the third type of conservative forces that must be taken into account in DPD simulations. Explicit electrostatics was incorporated in the DPD method by Groot et al. [49] and by others [50,51,52]. However, its implementation is not straightforward. First, the non-screened Coulomb potential (CP) describing the interaction between two point particles diverges at short distances. While this divergence is not a problem in bead-spring models employing the Lennard–Jones potential, the repulsive part of which diverges even more and prevents the collapse of the oppositely charged particles on top of each other, the use of the non-screened CP is prohibited in simulations which employ soft and non-diverging repulsive forces because the electrostatic forces would dominate over repulsive forces at shorts distances and cause undesired consequences. Several different approaches have been used to overcome this problem, e.g., the cutting-off the Coulomb potential at short distances, adding a small hard core or another steeply changing potential either in the center of DPD bead or around the bonds connecting DPD beads in the polymer chain [53]. The latter methods were used mainly in rheology studies to emulate the entanglements of long polymer chains [54]. We use the most common approach preventing the computational problems consisting of a slight delocalization of the charge within the DPD. This approach is inspired by two facts. First, one DPD bead represents several monomeric units which all of them can be charged. Second and farther more important fact, the electrostatic potential between slightly smeared charges is “soft” and does not diverge at short distances. The advantage of this approach consists of the fact that it does not require artificial modification of repulsion parameters.

Several types of smearing, e.g., the linearly or exponentially decreasing charge density from the bead center, Gaussian density profile, etc., have been proposed and tested [55]. We use the most often used Slater-type exponential distribution [52],

(5) $$f\left(r\right)=\frac{qe}{\pi \lambda^{3}}\exp\left(-\frac{2r}{\lambda }\right),$$

where *q* is the charge fraction, *e* is the electron charge and λ is the decay length of the charge (smearing constant). The interaction between charged particles *i* and *j* is then given as:

(6) $$u_{ij}^{\mathrm{el}}=\frac{q_{i}q_{j}}{r_{ij}}\lambda_{B}k_{B}T\left[1-\exp\left(-\frac{2r_{ij}}{\lambda }\right)\left(1+\frac{11r_{ij}}{8\lambda }+\frac{3r_{ij}^{2}}{4\lambda^{2}}+\frac{r_{ij}^{3}}{6\lambda^{3}}\right)\right],$$

where $\lambda_{B}=e^{2}/\left(4\pi \varepsilon_{0}\varepsilon_{r}k_{B}T\right)$ is the Bjerrum length ($\varepsilon_{0}$ is the dielectric constant of a vacuum and $\varepsilon_{r}$ is the relative permittivity of the reference medium), $q_{i}$ and $q_{j}$ are their electric charges and can be reasonably approximated by a simpler formula [55]:

(7) $$u_{ij}^{\mathrm{el}}=\frac{q_{i}q_{j}}{r_{ij}}\lambda_{B}k_{B}T\left[1-\left(1+\beta r_{ij}\right)\exp\left(-2\beta r_{ij}\right)\right],$$

where β=5/(8λ).

Analogously to our earlier studies [50,56,57], the long part of the electrostatic interaction is treated in this work by the Ewald summation [58].

## 3. Model

**Figure 1:**
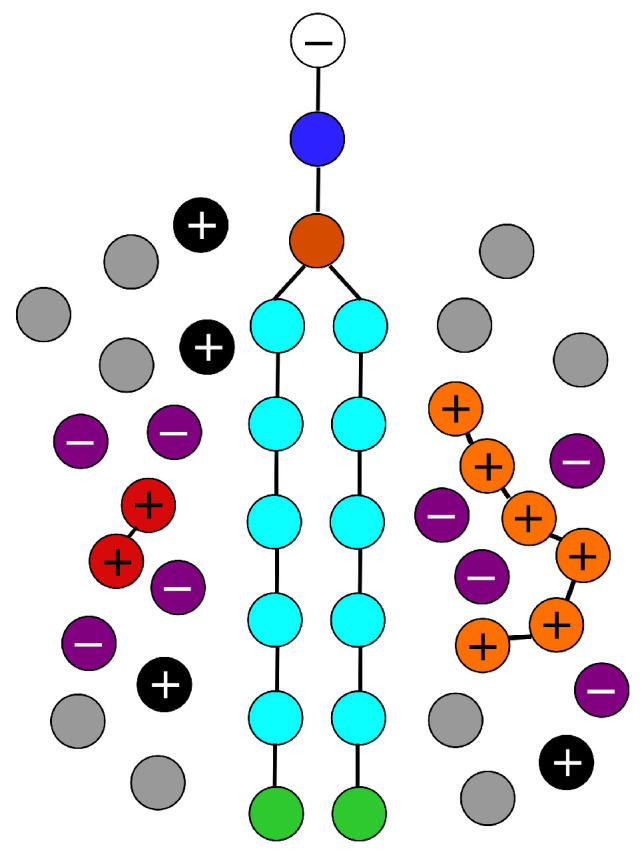
Negatively charged phospholipid head (Hq), white; electroneutral phospholipid head (H), blue; glycerol (G), dark orange; hydrophobic tail (C), cyan; end-bead of the tail (E), green; β-peptide (P), orange; monovalent cations (Na${}^{+}$), black; monovalent anions (Cl${}^{-}$), purple; divalent cations (Me${}^{2+}$), red; solvent particles (S), gray.

The membrane-forming phospholipids are modeled as the amphiphilic double-tail surfactants containing two chains formed by six strongly hydrophobic beads connected via one medium hydrophobic bead to a hydrophilic bead (see Figure 1). The interactions of phospholipids with other components are described by the commonly used parameters (see the next part). Their number (1708 phospholipids in a simulation box of the size $32^{3}$) is a result of a systematic preliminary study aimed at the reproducible formation of a regular continuous high-density membrane of appropriate lateral tension [59] preventing excessive bending (either spontaneous or interaction-induced bending). A great majority (90%) of phospholipids contain one neutral water-soluble (hydrophilic) terminal bead and 10% of species pose one negative elementary charge emulating the anionic group. For simplicity, none phospholipid bears any modifying group. The bonds are modeled by harmonic springs according to Equation (2) (k=128), the equilibrium distance $r_{0}=0.5$. and the angular cosine potential were applied to achieve the desired chain rigidity (θ=20).

The analogues of β-peptides are modeled as flexbile strings of six hydrophilic beads connected by harmonic springs. We performed two sets of simulations with 16 and 32 peptides adjusting the number of counterions to ensure the electroneutrality of the system.

The solvated monovalent positive ions (Na${}^{+}$) are represented by well-soluble beads (the same interaction parameters as for water-beads) bearing one positive elementary charge. Solvated divalent ions Me${}^{2+}$ (representing Ca${}^{2+}$ and/or Mg${}^{2+}$ ions) are modeled by two beads bound together by a very tough spring (k=250) with $r_{0}=0$. This means that the equilibrium distance between two beads is zero, i.e., they overlap and the mass of divalent ions is twice as large as that of monovalent ions. In simulations of systems with added salt, the solvated Cl${}^{-}$ anions are added (the same interaction parameters as for water-beads). In all simulations, the numbers of positive and negative species match and the systems are macroscopically electroneutral.

## 4. Parameter Setting

The formation, structure and properties of membranes formed by amphiphiles have already been studied by several researchers [60,61,62,63,64,65]. We follow the parameter setting used by Shillcock and Lipowsky [59] but we changed the value of the parameter describing the interaction of the hydrophobic group with water towards a slightly better solubility for the following reasons. Shillcock et al. used a relatively high value; $a_{\mathrm{HS}}=35$ for the hydrophilic group arguing that this group is less hydrophilic than, e.g., the solvated ions. This argument is relevant, but, based on our simulation studies of various amphiphilic and electrostatic self- and co-assembly of copolymers [50,56,57,66], we are of the opinion that this value is too high. Our studies of the self- and co-assembly of hydrophilic–hydrophobic diblocks A${}_{5}$B${}_{5}$ with a well-soluble block A and a worse soluble block B indicated that the value $a_{\mathrm{BS}}=35$ is a marginal value representing the solubility limit for A${}_{5}$B${}_{5}$ copolymers [56,57]. The copolymers with uncharged blocks are still “soluble” but the unimer fraction is fairly low and the fractions of various loose and fast temporarily fluctuating aggregates are high. Moreover, the well-defined and fairly uniform (low dispersity) multimolecular core-shell aggregates with compact inter-polyelectrolyte complex (IPEC) cores (a complex of B${}^{+}$ and B${}^{-}$ blocks) spontaneously form upon mixing the solutions of A${}_{5}$B${}_{5}$ copolymers with oppositely charged B-blocks (both described by $a_{\mathrm{BS}}=35$). Therefore, in our simulations we used a lower value $a_{\mathrm{HS}}=27$. The used parameters in our study are summarized in Table 1.

**Figure 2:**
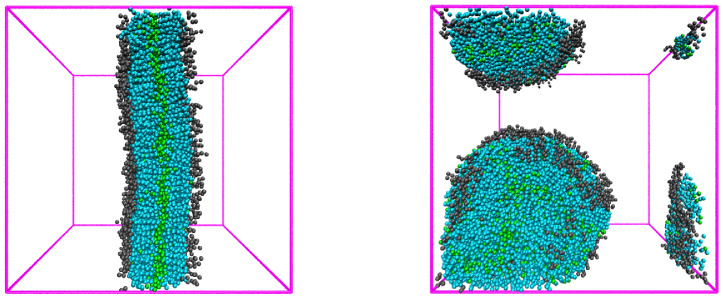
**Left**: The membrane formed in a $5\times 10^{9}$ step long simulation run in a box $32^{3}$ using the interaction $a_{\mathrm{HS}}=27$. **Right**: A typical self-assembly formed in a $10^{9}$ step long simulation run in a box $32^{3}$ using the interaction parameter $a_{\mathrm{HS}}=35$.

To acquire the confidence that this value properly describes the H-S interaction, we investigated the effect of $a_{\mathrm{HS}}$ in more detail. We performed a series of simulations with $a_{\mathrm{HS}}=27$ and 35 in simulation boxes of different sizes, from $25^{3}$ to $40^{3}$ increasing appropriately the number of amphiphiles (and other components) and the length of simulation runs. While the simulations with $a_{\mathrm{HS}}=27$ always produced well-arranged bilayer membranes, the simulations with $a_{\mathrm{HS}}=35$ generated the well-defined membranes across the simulation box only in the smallest box. In larger boxes, the simulations (even the excessively prolonged ones) resulted in quite irregular structures (in the best cases in patches of two bilayers with their “hydrophilic” surfaces tightly adhering to each other—see Figure 2). In brief, the simulations with a relatively low hydrophilicity of the head-group described by $a_{\mathrm{HS}}=35$ produce irregular self-assemblies with a number of misarranged units due to the fact that the difference between interactions parameters $a_{\mathrm{TS}}$ and $a_{\mathrm{TH}}$ is larger than the difference between parameters $a_{\mathrm{TH}}$ and $a_{\mathrm{HS}}$. This simply means that, in early stages, when the phospholipids are randomly molecularly dispersed in the aqueous medium, the change in the system configuration in which the unfavorable tail–solvent interaction is replaced by the tail-head interaction and simultaneously the fairly favorable head-solvent interaction is replaced by a slightly less favorable head–tail interaction, i.e., the configuration change enabling the process leading to 3D structures, is accompanied by the appreciable decrease in internal energy. Hence, from the energetic point of view, the creation of compact 3D structures is a quite favorable process that can lead to a relatively deep local energy minima and prevent the achievement of the global energy minimum and the formation of a regular 2D bilayer. In the case of a more hydrophilic head-group with $a_{\mathrm{HS}}=27$, the above differences of interaction parameters are almost the same and the global energy minimum is deeper. Therefore, the regular membrane is reproducibly formed. This, we believe, demonstrates the insufficiently low hydrophilicity of the head-group of phospholipids described by $a_{\mathrm{HS}}=35$.

## 5. Methodology of Simulations

First, we simulated the spontaneous self-assembly of phospholipids in the pool of water molecules containing the appropriate number of univalent positive counterions. The simulations in a box $32^{3}$ with periodic boundary conditions started from several random mixtures of all components composed of 1708 model double-tail amphiphiles representing the phospholipids (90% neutral and 10% negatively charged, containing 171 charged phospholipid headgroups), 171 monovalent cations and 72,513 solvent molecules in a $32^{3}$ box and took $6\times 10^{10}$ simulation steps. After the equilibration period of $10^{10}$, we ran $5\times 10^{10}$ time steps. Repeated simulations always provided virtually identical flat 2D bilayers, which proves that the simulations are ergodic, do not freeze in arrested states, meet all other requirements of proper MD simulations and produce the equilibrium data.

Technical comment: The number of phospholipids in a box of a given size (based on careful preliminary studies) secures the formation of a compact continuous membrane of required physical properties (see the previous part) across the whole box oriented approximately perpendicular to four walls and parallel to the two remaining walls. Nevertheless, the membrane does not usually divide the box into two parts of the same volume. To simplify the evaluation of concentration profiles and other characteristics, the position of the basic simulation box was re-adjusted to receive almost equal volumes at both sides of the membrane.

Second, we performed simulations in systems with added small cations and anions. We observed that the addition of ions to the membrane formed in the first step yields a virtually identical equilibrium membrane to that obtained by the simulation starting with a random mixture of all components. The former variant shortens the necessary re-equilibration period and improves the quality of simulation data. Therefore, we repeatedly added increasing amounts of uni-univalent and di-univalent salts at random at both sides of the equilibrated membrane and performed new simulation runs at several ion concentrations. We always added the same amounts of ions at each side of the membrane. Even though the periodic boundary conditions secure the motion of ions within the whole simulation box, the addition of equal amounts of ions at each side of the membrane non-negligibly accelerates the equilibration.

Third, we performed the simulations with added positively charged oligomers (either 16 or 32). Based on the results of simulations of the membrane with added ions, we added the positively charged shorts chains into the box containing the equilibrium membrane and ions (one half of oligomer chains at each side).

Table 2 outlines the list of performed simulations and provides the numbers of molecules of individual components used in simulations.

## 6. Results and Discussion

### 6.1. Partially Charged Membrane with Monovalent and Divalent Cations

**Figure 3:**
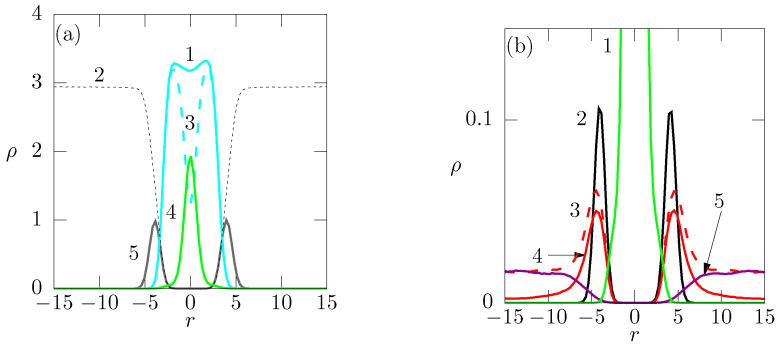
(**a**) Density profiles of phospholipid bilayer (system No. 1, see Table 2): 1 (cyan solid)—profiles of hydrophobic lipid tail, 2 (gray dashed)—solvent profile, 3 (cyan dashed)—hydrophobic beads (except the end beads), 4 (green solid)—noncharged end-bead profile, 5 (gray solid)—noncharged lipid heads profile; (**b**) Density profiles of charged end-groups and monovalent ions in the same systems No. 1 and 2 (Table 2): 1 (green solid)—noncharged end-bead profile, 2 (black solid)—negatively charged lipid head profile, 3 (red solid)—monovalent cations Na (20%), 4 (red dashed)—monovalent cations (10%), 5 (purple solid)—monovalent anions Cl (20%).

The simulations of the ensemble of 1708 double-tailed lipids in the $32^{3}$$r_{c}^{3}$ box yield reproducibly a compact membrane spanning across the $32^{2}$$r_{c}^{2}$ surface area of the simulation box. This means that the surface area per one head group is 0.6 $r_{c}^{2}$. The volume of one head group is by definition 1 $r_{c}^{3}$ and the volume of the whole double-tail phospholipid unit is 15 $r_{c}^{3}$, i.e., the surface-to-volume area per head is 0.6 $r_{c}^{-1}$. The compact membrane structure is demonstrated by Figure 3, which shows the formation of a regular phospholipid bilayer. It shows the ensemble-average density profiles of individual components as functions of the perpendicular distance from the hydrophobic center of the bilayer averaged over $10^{10}$ simulation steps and in every simulation step over the whole surface of the membrane. In the case of a slightly curved membrane, the local perpendicular directions are defined with respect to the actual tangent line at a given position to the curve connecting the membrane centers.

In Figure 3a, the density profiles of all components are shown (including the solvent). The density of hydrophobic beads is high which proves that the membrane is compact. The shape of the T-curve (3, dashed cyan) depicting the density of the hydrophobic beads (except the end beads) with a pronounced minimum in the center confirms the bilayer structure. The shape of the E-curve (4, green solid) indicates that the hydrophobic central beads are localized in a fairly narrow region. However, its halfwidth is non-negligible in comparison with the total width of the membrane, which suggests that the positions of relatively flexible ends of chains fluctuate. The sum of T- and E-curves (1, cyan solid) passes a shallow local minimum at r=0 which reflects a slightly increased mobility of end-segments and is consistent with the non-negligibly broad distribution of their positions. As the numbers and consequently the density profiles of charged species, and particularly these positively charged monovalent ions, are low, Figure 3b depicts the vertical-axis-enlarged profiles of charged end-groups and of ions only. A part of the concentration profile of central end-beads is shown for a comparison of the widths of concentration profiles of minority and majority components. In the case of pure membrane (without added salts), the positively charged counterions efficiently compensate the negative charge of the membrane at short distances and only a low fraction of free ions move in the bulk solvent. Note that the univalent ions are the only positively charged species present in the system and their number is the same as that of negatively charged phospholipids embedded in the membrane. The addition of the uni-univalent salt increases the concentration of positively charged ions in the vicinity of the membrane only slightly because the negative charge has already been compensated for by counterions and the majority of ions (both positive and negative) uniformly spread in bulk. As expected, the negative ions are repealed by the negatively charged membrane and their concentration in the immediate vicinity of the membrane decreases to zero.

**Figure 4:**
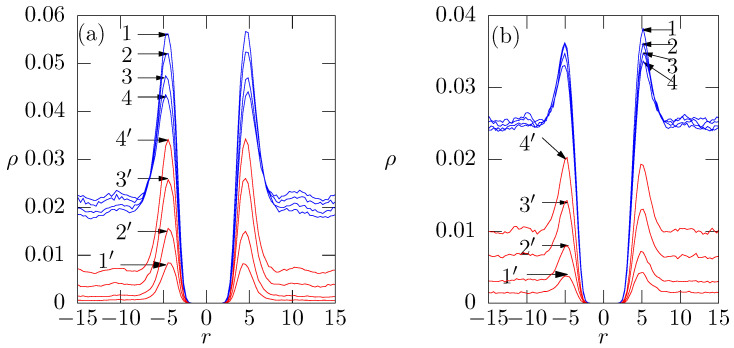
(**a**) Concentration profiles of monovalent and divalent ions added to the membrane; (**b**) Change in concentration profiles of ions after addition of the positively charged oligomer chains. In (**a**), curves (1), (2), (3) and (4) corresponds to concentration of monovalent cations in systems with increasing amount of Me${}^{2+}$ from 25, 50, 100 to 150; Curves $\left(1^{\prime }\right),\left(2^{\prime }\right),\left(3^{\prime }\right)$ and $\left(4^{\prime }\right)$ corresponds to Me${}^{2+}$ added to the membrane. In (**b**), curves (1), (2), (3), (4) corresponds to monovalent cations, curves $\left(1^{\prime }\right),\left(2^{\prime }\right),\left(3^{\prime }\right)$ and $\left(4^{\prime }\right)$ to Me${}^{2+}$ with 32 added oligomer chains.

The next part addresses the competitive binding of univalent and divalent ions to the negatively charged membrane. Figure 4a depicts the changes in the spatial concentration of uni- and divalent positive ions during the stepwise addition of their 2:1 mixture (i.e., 1:1 mixture concerning the charges) to the equilibrated membrane, the charge of which has already been compensated for by univalent counterions. For easy understanding of the curves shown in Figure 4 and for smooth discussion of their changes upon the addition of salts, we would like to stress the following fact: the electroneutrality of the system containing the neat membrane (without added salts) requires the presence of 171 Na${}^{+}$ cations in the simulation box. As shown in Figure 3b, almost all of the Na${}^{+}$ ions concentrate in the vicinity of the negatively charged membrane and compensate its charge and therefore their concentration in bulk solvent (at high *r*) is very low. In the next part of the study, we gradually added the mixtures containing (i) 50 Na${}^{+}$ and 25 Me${}^{2+}$, (ii) 100 Na${}^{+}$ and 50 Me${}^{2+}$, (iii) 200 Na${}^{+}$ and 100 Me${}^{2+}$ and finally (iv) 300 Na${}^{+}$ and 150 Me${}^{2+}$ ions and the corresponding number of Cl${}^{-}$ anions, which means that the blue curves correspond to the Na${}^{+}$ numbers ranging from 221 to 471 and the red ones to Me${}^{2+}$ numbers ranging only from 25 to 150. The difference in concentrations of monovalent and divalent ions explains why the blue curves corresponding to Na${}^{+}$ are higher than the red curves corresponding to Me${}^{2+}$. Nevertheless, the changes in concentration profiles clearly prove the preferential electrostatic binding of divalent ions to the membrane. The peaks maxima at ca. r=5 at the red curves increase with the increase in the amount of added Me${}^{2+}$, while those at blue curves decrease. This indicates that the monovalent ions in the immediate vicinity of the membrane are replaced by divalent ions and liberated into the bulk solution, which is witnessed by gradually increasing plateau concentrations at long distances from the membrane. A very important message ensues from the comparison of concentrations of ions in bulk. Remarkably low bulk concentrations of Me${}^{2+}$ indicate that the major part of divalent ions concentrate close to the membrane and only a minor part spreads in bulk solvent which in turn mainly contains the univalent ions. The preferential binding of Me${}^{2+}$ ions is promoted both by enthalpy and by entropy. The electrostatic attraction of double-charged ions at short distances from the membrane is stronger than that of single-charged ions and the translational entropy of fairly massive double charged ions, such as Ca${}^{2+}$ decreases less upon their localization in a narrow region in the membrane vicinity than that of lighter and hence more mobile single-charged ions.

### 6.2. Effect of the Binding of Positively Charged Oligomers at the Membrane on the Distributions of Univalent and Divalent Ions

The most important part of the study concerns the effect of positively charged soluble oligomer chains (model of short β-peptides) on the spatial distribution of ions and particularly on the changes in density profiles of the double charged ions in the closest neighborhood of the membrane. Because the interaction of Me${}^{2+}$ (Mg${}^{2+}$ and/or Ca${}^{2+}$) with the membrane embedded receptors and channels is essential for the function of bacteria, the results of simulations can support or disprove the working hypothesis proposed by Qian et al. [14].

**Figure 5:**
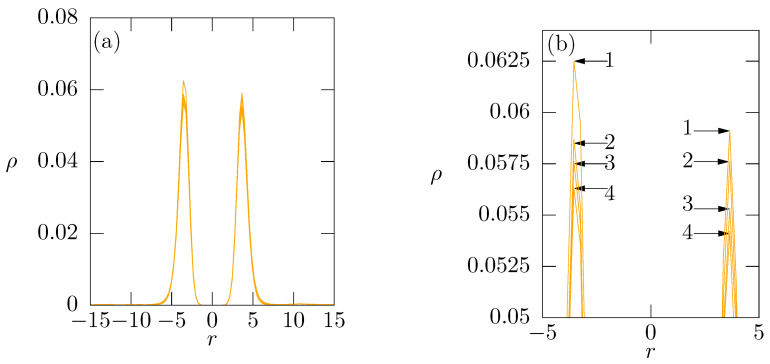
(**a**) Density profiles of 32 oligomer β-peptides added to the membrane; (**b**) Zoom of peaks of density profiles of oligomer β-peptides of amount of 32. Curve (1), (2), (3), (4) corresponds to 25, 50, 100, 150 Me${}^{2+}$ beads respectively.

The simulation results are summarized in Figure 4b where, for simplicity and clarity of presentation, we depict only the concentration of physiologically relevant cations. From the comparison of concentration profiles in Figure 4a,b, we immediately see that the positively charged oligomer chains replace the ions compensating the membrane charge. The peaks of Me${}^{2+}$ (see Figure 4) at ca. r=5 decrease significantly upon the addition of oligomer chains and the plateau regions at high *r* slightly increase. Analogously to the behavior discussed in the previous paragraph, the release of ions is driven by the synergy of enthalpy and entropy. In this case, the entropy driven process is reinforced by the cooperative electrostatic effect of multiple charges on the oligomer chains which results in the strong binding of added oligomer chains to the membrane surface as witnessed by narrow density profiles of fixed chains unaffected by changes in ions concentration (Figure 5).

The authors of the biomedical study observed a partial recovery of the activity of bacteria treated with β-peptide analogues after the addition of Mg${}^{2+}$ and Ca${}^{2+}$ salts. Our simulations performed at medium ion concentrations relevant for biochemical studies did not reveal a remarkable effect of added salts. However, as stated in the Introduction, we do not aim at the accurate reproduction of the experiments performed by Qian et al., but at general features of the behavior of systems containing the membrane, charged oligomers and ions. Our study does not emulate the incubation of bacteria at surfaces covered by tethered β-peptide analogues followed by the addition of salts. The main differences between our simulation study and the motivation paper are the following: (i) The oligomer chains added in our simulation are molecularly soluble and firmly adhere to the membrane surface, while the analogues of β-peptides covalently attached to the gold surface can be mechanically removed after the incubation; (ii) Our model chains are slightly more charged than the synthetic analogues of the β-peptides; (iii) In our simulations, we discern only between the single- and the double-charged ions and neglect the fact that the mass of Mg${}^{2+}$ is approximately one half of that of Ca${}^{2+}$ and the translational entropy of Mg${}^{2+}$ and all consequent entropy effects are more important. With respect to the above arguments, we restrain from any comments on the effect of the ions added after the treatment of bacteria with the surface-tethered β-peptide analogues. Nevertheless, the results and conclusion concerning the impact of salts on the electrostatically attached β-peptide analogues observed in our simulation study are relevant. The simulation data show that the binding of positively charged oligomers is strong and the results do not depend on the sequence how the components are added (results not shown—data available upon request).

The simulated density profiles at both sides of the membrane obtained in several repetitions of the long simulation run ($10^{10}$ simulation steps) are slightly asymmetrical. The differences correspond to the ±1 chain only, but we are of the opinion that this fact needs to be discussed and explained. We believe that a small asymmetry of peaks at the opposite sides of the membrane is the result of a strong local electrostatic binding. Even though the initial configurations of all components (including the negatively charged headgroups at the membrane) are random and more or less uniform and the periodic boundary conditions secure free motion of β-peptide analogues within the whole box, we cannot preclude that β-peptide analogues can be trapped in spots with an increased local negative charge density during the whole run, while the overwhelming part of the system undergoes a proper equilibration. As the asymmetry corresponds to one or max two chains from 32, we are of the opinion that it does not invalidate the simulation results. Moreover, the simulation data (available upon request) show that the asymmetry of peaks decreases with the number of added chains and also with increasing ionic strength because the added ions screen and efficiently weaken the electrostatic interaction.

**Figure 6:**
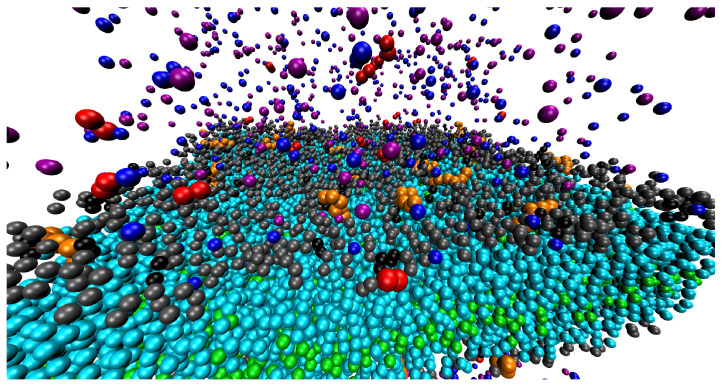
Snapshot from simulation with 50 divalent cations and 32 β-peptides. Negatively charged phospholipid head (Hq), black; electroneutral phospholipid head (H), gray; glycerol (G), yellow; hydrophobic tail (C), cyan; end-bead of the tail (E), green; β-peptide (P), orange; monovalent cations (Na${}^{+}$), blue; monovalent anions (Cl${}^{-}$), purple; divalent cations (Me${}^{2+}$), red; solvent particles (S), white.

To give the reader a clear idea of the studied system and the binding of analogues of β-peptide at the membrane, in Figure 6 we show a typical snapshot of the membrane. It is obvious that the positively charged oligomers (analogues of β-peptides) firmly stick to the negatively charged membrane replacing the small ions. However, a non-negligible number of ions (mainly the divalent ones) can be found close to the membrane, i.e., the oligomers (in spite that their positive charge exceeds that of negative groups at the membrane) do not block the approach of positive ions to the membrane. The reason is clear: the close localization of several charges at their oligomers promotes the electrostatic binding to the membrane, but it leaves large parts of the membrane electrostatically unaffected by the oligomers.

We cannot preclude that the effect observed by Qian et al. [14] in a complex biological system could have been affected and somewhat modified by specific biochemically relevant interactions. Nevertheless, we can, without any doubts, confirm that the tentative hypothesis proposed by Qian is sound and explains at the molecular level the mechanism that decisively contributes. Our study elucidates at the molecular level the mechanism that contributes to the suppression of gram-negative bacteria activity upon the interaction with the surface-tethered analogues of β-peptides.

## 7. Summary and Conclusions

The performed simulations show that the positively charged divalent ions preferentially bind to partially (10%) negatively charged phospholipid bilayer membranes in solutions containing the mixtures of monovalent and divalent ions not only because they are double charged and their interaction at short distances is strong but because their mass is higher and their mobility and consequently their translational entropy are lower and hence their localization close to the membrane does not decrease the overall entropy of the system as much as that of the mobile monovalent ions.

The ions compensating the membrane charge can by efficiently liberated and replaced by multiply charged oligomeric chains or by short polyelectrolytes, e.g., by short positively charged β-peptides and by their synthetic analogues as observed and studied experimentally by Qian [14]. The binding of multiply charged oligomers and that of ions are the competing processes controlled by concentrations of corresponding species but the strong electrostatic interaction supported by the synergic entropy contribution favors the binding of charged oligomers and prevents their liberation from the membrane surface upon the increase in ion concentration within the limits of concentrations relevant for functional biological systems.

The charged oligomer chains sticking firmly to the membrane cause a partial depletion of the concentration of inorganic ions in the water-membrane interfacial region, but they do not prevent the approach of some ions to the membrane surface at their elevated concentrations as witnessed by peaks in ion concentration close to the membrane surface, which partially diminish, but do not disappear. Our simulation results qualitatively agree with all experimental observations published by Qian et al. and corroborate their explanatory hypothesis. They contribute to studies aimed at the biological impact of β-peptides on the gram-negative bacteria [30,31,32,33,34,35,36] and deepen the knowledge on the mechanism of their action.

From the viewpoint of the DPD simulation method, the results generally confirm the numeric values of interaction parameters proposed by Shillcock et al. [59] for the membrane-forming species, except one. The simulation study of the box-size effect shows that the interaction parameter $a_{\mathrm{HS}}=35$ does not guarantee the formation of a regular bilayer. The observation that the 2D membrane is reproducibly formed in the small 25${}^{3}$ box, but fails to form in larger boxes (32${}^{3}$ and 40${}^{3}$) is interesting. We believe that this behavior can be explained by the following arguments. As already discussed at page 9, the formation of irregular 3D associates is accompanied by significant decrease in internal energy. As the local energy minimum is deep, the reconstruction of compact 3D associates into the 2D bilayer is difficult, because the activation energy necessary for surmounting the energy barrier between the local and the global minimum is high. The larger the simulation box, the larger the 3D associates that form and the more and more difficult the transition. Despite observing the significant changes in the size and in shape of irregular associates in large boxes during the excessively long simulation runs (i.e., the simulation does not totally freeze), the simulation trajectories suggest that the system is unable to escape from the deep local minimum.

## Figures and Tables

**Table 1:** Interaction parameters $a_{ij}$, dissipative coefficients $\gamma_{ij}$ [53]. H—electroneutral phospholipid head, Hq—negatively charged phospholipid head, G—glycerol, T—hydrophobic tail, E—end-bead of the tail, P—β-peptide, Na${}^{+}$—monovalent cations, Cl${}^{-}$—monovalent anions, Me${}^{2+}$– divalent cations, S—solvent particles.

	$a_{\mathrm{ij}}$/$\gamma_{\mathrm{ij}}$							
	**H**	**Hq**	**G**	**T, E**	**S**	**Na${}^{+}$/Cl${}^{-}$**	**Me${}^{2+}$**	**P**
H	25/4.5	25/4.5	27/4.5	50/9.0	27/4.5	27/4.5	25/4.5	27/4.5
Hq		25/4.5	27/4.5	50/9.0	27/4.5	27/4.5	27/4.5	27.4/5
G			25/4.5	50/9.0	28/4.5	28/4.5	28/4.5	28/4.5
T, E				25/4.5	75/20.0	75/20.0	75/20.0	27/4.5
S					25/4.5	25/4.5	25/4.5	25/4.5
Na${}^{+}$/Cl${}^{-}$						25/4.5	25/4.5	25/4.5
Me${}^{2+}$							25/4.5	25/4.5
P								25/4.5

**Table 2:** The list of performed simulations in a box of volume $32^{3}$ with the total number of monovalent cations Na${}^{+}$, monovalent anions Cl${}^{-}$, divalent cations Me${}^{2+}$ and β-peptides P.

Membrane with	Me${}^{2+}$	P	Na${}^{+}$/Cl${}^{-}$	Membrane with	Me${}^{2+}$	P	Na${}^{+}$/Cl${}^{-}$
1. 10% Na	0	0	171/0	9. salt + 25 Me${}^{2+}$ + 16 P	25	16	584/413
2. 20% Na${}^{+}$ 10% Cl${}^{-}$	0	0	342/171	10. salt + 25 Me${}^{2+}$ + 32 P	25	32	538/367
3. salt + 25 Me${}^{2+}$	25	0	392/221	11. salt + 50 Me${}^{2+}$ + 16 P	50	16	634/463
4. salt + 50 Me${}^{2+}$	50	0	442/271	12. salt + 50 Me${}^{2+}$ + 32 P	50	32	638/467
5. salt + 100 Me${}^{2+}$	100	0	542/371	13. salt + 100 Me${}^{2+}$ + 16 P	100	16	734/564
6. salt + 150 Me${}^{2+}$	150	0	642/471	14. salt + 100 Me${}^{2+}$ + 32 P	100	32	738/567
7. salt + 25 Me${}^{2+}$ + 16 P	25	16	488/317	15. salt + 150 Me${}^{2+}$ + 16 P	150	16	738/567
8. salt + 25 Me${}^{2+}$ + 32 P	25	32	584/413	16. salt + 150 Me${}^{2+}$ + 32 P	150	32	834/663

## Data Availability

The data presented in this study are available upon request from the corresponding authors.

## Abbreviations

The following abbreviations are used in this manuscript:

MDPI	Multidisciplinary Digital Publishing Institute
MRSA	Methicillin-resistant *Staphylococcus aureus*
DPD	Dissipative particle dynamics
FENE	Finitely extendible non-linear elastic
FH	Flory-Huggins
CP	Coulomb potential
IPEC	inter-polyelectrolyte complex
MD	Molecular Dynamics
